# TRPV3 and TRPV4 ion channels are not major contributors to mouse heat sensation

**DOI:** 10.1186/1744-8069-7-37

**Published:** 2011-05-17

**Authors:** Susan M Huang, Xiaoxin Li, YinYin Yu, Juan Wang, Michael J Caterina

**Affiliations:** 1 Department of Biological Chemistry, Department of Neuroscience, Center for Sensory Biology, Johns Hopkins School of Medicine, Baltimore, MD USA

## Abstract

**Background:**

The discovery of heat-sensitive Transient Receptor Potential Vanilloid (TRPV) ion channels provided a potential molecular explanation for the perception of innocuous and noxious heat stimuli. TRPV1 has a significant role in acute heat nociception and inflammatory heat hyperalgesia. Yet, substantial innocuous and noxious heat sensitivity remains in TRPV1 knockout animals. Here we investigated the role of two related channels, TRPV3 and TRPV4, in these capacities. We studied TRPV3 knockout animals on both C57BL6 and 129S6 backgrounds, as well as animals deficient in both TRPV3 and TRPV4 on a C57BL6 background. Additionally, we assessed the contributions of TRPV3 and TRPV4 to acute heat nociception and inflammatory heat hyperalgesia during inhibition of TRPV1.

**Results:**

TRPV3 knockout mice on the C57BL6 background exhibited no obvious alterations in thermal preference behavior. On the 129S6 background, absence of TRPV3 resulted in a more restrictive range of occupancy centered around cooler floor temperatures. TRPV3 knockout mice showed no deficits in acute heat nociception on either background. Mice deficient in both TRPV3 and TRPV4 on a C57BL6 background showed thermal preference behavior similar to wild-type controls on the thermal gradient, and little or no change in acute heat nociception or inflammatory heat hyperalgesia. Masking of TRPV1 by the TRPV1 antagonist JNJ-17203212 did not reveal differences between C57BL6 animals deficient in TRPV3 and TRPV4, compared to their wild-type counterparts.

**Conclusions:**

Our results support the notion that TRPV3 and TRPV4 likely make limited and strain-dependent contributions to innocuous warm temperature perception or noxious heat sensation, even when TRPV1 is masked. These findings imply the existence of other significant mechanisms for heat perception.

## Background

TRPV1 is a non-selective cation channel that can be activated by heat (at > ~42°C) or a wide range of chemical agonists such as capsaicin and acid [1]. TRPV1 is highly expressed in small diameter primary sensory neurons. Mice deficient for TRPV1 show blunted noxious heat perception in tests of acute heat nociception and inflammatory heat hyperalgesia [2]. Although these responses are impaired, mice devoid of TRPV1 are still able to respond to heat. For example, although TRPV1 knockout mice exhibit a 4-fold longer tail withdrawal latency at 50°C, they still withdraw their tails in response to hot water. In one TRPV1 knockout line, behavioral deficits were reported in thermal hyperalgesia but not in acute heat nociception [3]. Moreover, thermal selection behavior on a thermal gradient is normal in the absence of TRPV1 [4]. Significant residual responses to heat have also been observed in skin-nerve explants derived from TRPV1 knockout mice [2,5,6]. Thus, other mechanisms must exist for the perception of innocuous and noxious heat. Among the most promising candidate mediators of TRPV1-independent heat sensation are homologous members of the TRPV subfamily that can also be activated by warm/hot temperatures (TRPV4 at > 27°C, TRPV3 at > 33°C, TRPV2 at > 52°C) [7-13].

TRPV4 is expressed in a wide range of tissues including primary sensory neurons and skin keratinocytes [8,9]. Keratinocytes from TRPV4 knockout animals lack TRPV4-like warmth-evoked currents [14]. Nerve fiber recordings have suggested that warmth-evoked electrical activity may be diminished in TRPV4 knockout mice [15]. Under naïve conditions, mice deficient for TRPV4 have been reported to exhibit slightly prolonged withdrawal latencies to moderately hot temperatures in the tail immersion assay, but no differences from wild-type were seen in the hot plate or radiant paw-heating assays [15-18]. However, their escape latencies in the hot plate test were reported to be longer than those of wild-type mice in the context of inflammation [15]. In assays of innocuous thermal perception, we previously observed that TRPV4 knockout mice prefer slightly warmer temperatures compared to wild-type littermates in both thermal gradient and two-temperature choice paradigms [16].

TRPV3 is another heat-sensitive channel that is expressed prominently in rodent skin keratinocytes [10], though it may be expressed in neurons as well [11,13]. Wild-type keratinocytes exhibit warmth-evoked currents that are distinct from TRPV4-like currents [14]and are absent upon TRPV3 gene disruption [19]. Mice deficient for TRPV3 were reported to select preferred floor temperature at a slower pace but settle in the same temperature range as wild-type littermates [19] and to have a decreased preference for 35°C over room temperature in a two-temperature choice paradigm. In tests of acute heat nociception, longer withdrawal latencies were observed at high temperatures [19]. TRPV3 knockout keratinocytes were also reported to be deficient in heat-evoked release of the pronociceptive molecule, ATP [20].

One potential complication of studies investigating the roles of TRPV3 and TRPV4 in heat sensation is that the temperature-response curves of these two channels overlap in the innocuous warmth range. It is therefore possible that the behavioral consequences of gene disruption of one channel are compensated by the other channel. Moreover, since TRPV1 is known to have a prominent role in heat perception, it is possible that the presence of this channel masks contributions from TRPV3 and/or TRPV4. Therefore we set out to study whether heat sensation would be altered by genetically disrupting both TRPV3 and TRPV4, with and without simultaneous functional masking of TRPV1.

## Results

### Heat Sensitivity in TRPV3 Knockout Mice on the C57BL6 Background

**Figure 1:**
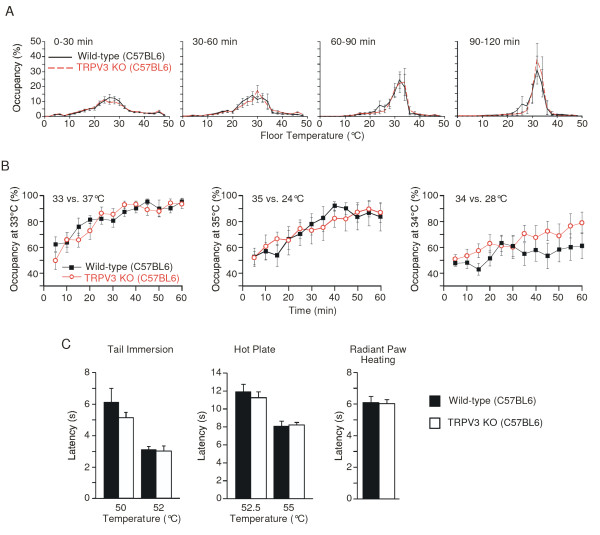
**Temperature preference and heat-evoked acute nociceptive behavior of TRPV3 knockout mice on the C57BL6 background**. (A) Thermal preference behavior of freely moving wild-type (black solid line, n = 12) and TRPV3 knockout (KO, red dashed line, n = 12) mice on a floor temperature gradient of 0.8°C to 48.8°C over 120 minutes. Percent of time spent (mean ± SEM) within the indicated 2°C bins was monitored during the indicated time periods. (B) Thermal preference behavior of freely moving wild-type (filled black squares, n = 10-12) and TRPV3 knockout (KO, open red circles, n = 10-12) mice in a two-temperature selection task. Percent of time spent (mean ± SEM) at the indicated floor temperature was monitored for 5 min intervals over 60 min. Floor temperature choices were 33°C vs 37°C (left), 35°C vs. 24°C (middle), and 34°C vs. 28°C (right). (C) Heat-evoked acute nociceptive behavior of wild-type (filled bars) and TRPV3 KO (open bars) mice. Latency to response was measured in the tail immersion (left, n = 13) and hot plate (middle, n = 10) assays at the indicated temperatures. Latency to response in the radiant paw heating assay (n = 13-14) was measured during stimulation at a fixed lamp intensity. Data represent mean ± SEM.

The previously published analysis of heat sensation in TRPV3 knockout mice was conducted on a randomly mixed C57BL6 and 129J N1 background [19]. Because genetic background can strongly influence thermal nociceptive behavior [21], and to simplify crosses with TRPV4 knockout mice which were on a C57BL6 background, we began our experiments by analyzing TRPV3-deficient mice [19] that had been backcrossed 8 generations onto a C57BL6 background (gift of Dr. Ardem Patapoutian). Wild-type and TRPV3 knockout C57BL6 mice showed similar temperature selection behaviors on a floor gradient spanning from 0.8 to 48.8°C. In both cases, the mice settled in the region of the gradient around 32°C over a 2 hr period. Unlike the phenotype reported on the mixed genetic background, however, selection behaviors were virtually superimposable between genotypes in each 30 minute period of the assay (Figure 1A). We also observed no major differences between TRPV3 knockout mice and wild-type controls in a two-temperature selection assay. The two genotypes exhibited similar avoidance of innocuous warm temperature (preferring 33°C over 37°C) and avoidance of innocuous cool temperature (preferring 35°C over 24°C). Even in a more difficult discrimination task with the test temperatures closer together (34°C versus 28°C), the performance between the two genotypes was similar (Figure 1B). Although there was a slight overall increased preference of TRPV3 knockout mice for 34°C in this task (wild-type vs. knockout: overall *p *< 0.01, *n *= 13, two-way ANOVA with Bonferroni correction for repeated measures), this difference was not statistically significant at any single time point.

We next assayed acute heat pain behavior in TRPV3 knockout mice on a C57BL6 background. These mice exhibited withdrawal responses that were no different from wild-type controls in the tail immersion assay (50°C, 52°C), the hot plate assay (52.5°C, 55°C) or the radiant paw-heating assay (Figure 1C). Thus, TRPV3 does not appear to be essential for acute thermal nociception in C57BL6 mice.

### Heat Sensitivity in TRPV3 Knockout Mice on the 129S6 Background

**Figure 2:**
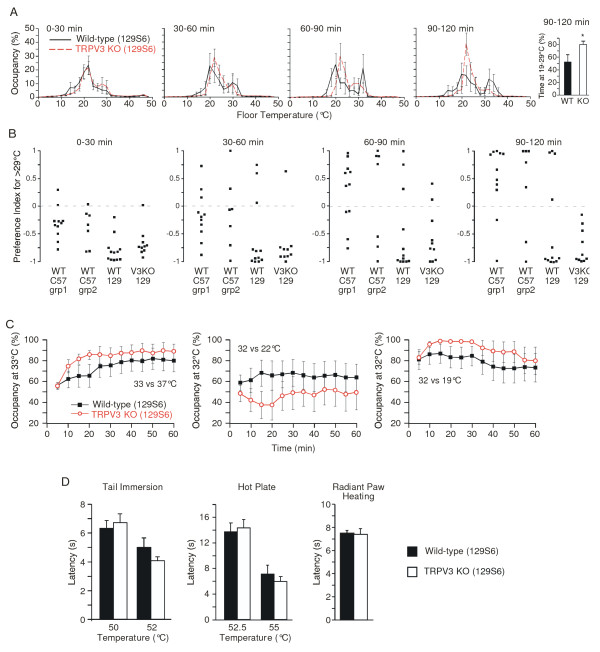
**Temperature preference and heat-evoked nociceptive behavior of TRPV3 knockout mice on the 129S6 background**. (A) Thermal preference behavior of wild-type (black solid line, n = 12) and TRPV3 knockout (KO, red dashed line, n = 10) mice on a floor temperature gradient of 0.8 to 48.8°C. Percent of time (mean ± SEM) spent within the indicated 2°C bins was monitored during the indicated time periods. Right panel shows percent of time (mean ± SEM) spent by wild-type (filled bar) and knockout (open bar) mice between 19°C and 29°C during the final 30 min. (B) Preference index of individual mice for temperatures > 29°C in the thermal gradient. Wild-type C57BL6 mice were those presented in Figure 1A (grp 1, n = 11) or a separate group (grp 2, n = 7) assayed at the same time as the 129S6 mice from Figure 2A (129S6 wild-type n = 12, 129S6 TRPV3 KO n = 10). (C) Thermal preference of wild-type (filled black squares, n = 10-12) and TRPV3 KO (open red circles, n = 10-12) mice in two-temperature selection tasks. Percent of time spent (mean ± SEM) at the indicated temperature was monitored at 5 min intervals. (D) Heat-evoked acute nociceptive behavior of wild-type (filled bars, n = 12) and TRPV3 KO (open bars, n = 10) mice. Latency to response was measured in the tail immersion (left), hot plate (middle), and radiant paw heating (right) assays. Data represent mean ± SEM. (* p < 0.05, wild-type vs. knockout, unpaired t-test).

The differences between our data and those previously reported for TRPV3 knockout mice on the mixed genetic background prompted us to evaluate whether the contribution of TRPV3 to thermal nociception might be more pronounced on a non-C57BL6 background. We therefore backcrossed the TRPV3 null allele over six generations onto the 129S6 background and intercrossed the resulting offspring to generate wild-type and TRPV3 knockout 129S6 experimental mice. In the thermal gradient assay, we found that, unlike wild-type C57BL6 mice, which exhibit a unimodal distribution that gradually develops a prominent peak at ~32°C (Figure 1A), wild-type 129S6 mice exhibited a more complex thermal preference pattern. Whereas mean occupancy during the first 30 min was relatively unimodal, with a peak near ~22°C, over the ensuing 90 min, their distribution became more bimodal, with roughly equivalent peaks around ~22°C and ~32°C (Figure 2A). In TRPV3 knockout 129S6 mice, the distribution during the initial 30 minutes was similar to that of wild-type 129S6 controls. However as the assay progressed, occupancy near ~22°C became more pronounced, such that these mice spent more time between 19°C and 29°C, compared with wild-type controls (Figure 2A, right two panels). In principle, the bimodal distribution of the wild-type 129S6 mice could result either from individual mice spending part of their time in either temperature range or from some of the mice spending a majority of their time in one range and others spending a majority of their time in the other range. To distinguish these possibilities, we calculated a preference index for time spent either above or below 29°C in four groups of mice: wild-type 129S6 mice, TRPV3 knockout 129S6 mice, wild-type C57BL6 mice from a prior experiment described in Figure 1, and a separate, smaller cohort of wild-type C57BL6 mice assayed at the same time as the 129S6 mice (Figure 2B). In both groups of C57BL6 mice, most individuals occupy temperatures < 29°C at the outset of the assay, but over time, the majority of these mice exhibit a greater preference for temperatures > 29°C. In contrast, whereas a few wild-type 129S6 mice gradually develop a strong preference for temperatures > 29°C, the majority of these mice strongly favor temperatures < 29°C. Thus, the bimodal character of the wild-type 129S6 distribution is apparently attributable to heterogeneous direction of strong preference among individual mice, rather than to a given mouse equally occupying two temperature ranges. In the TRPV3 knockout 129S6 group, the greater occupancy at cooler temperatures results from approximately half of the animals exhibiting a strong preference for temperatures < 29°C, and the other half exhibit a weaker preference for < 29°C.

To further examine the possibility of a thermal selection phenotype in TRPV3 knockout 129S6 mice, we utilized the two-temperature preference assay. TRPV3 knockout 129S6 mice showed a slightly greater mean preference than wild-type controls for 33°C over 37°C, and less preference than controls for 32°C over 22°C (wild-type vs. knockout: overall *p *< 0.01 in both comparisons, *n *= 10-12, two-way ANOVA with Bonferroni correction for repeated measures), although these differences did not reach statistical significance at any single time point (Figure 2C). In the 32°C vs. 22°C selection task, which was designed based upon the two occupancy peaks observed on the thermal gradient assay, thermal selection was highly variable, with multiple mice of each genotype spending most of their time at the "nonpreferred" temperature. To determine whether this small apparent difference between genotypes could be amplified, we assayed occupancy at 32°C vs. 19°C. Although mice of both genotypes preferred 32°C, the overall preference of TRPV3 knockout mice for this warmer temperature was slightly greater than that of wild-type controls (wild-type vs. knockout: overall *p *< 0.01, *n *= 10-12, two-way ANOVA with Bonferroni correction for repeated measures). Again, these differences did not reach statistical significance at any single time point (Figure 2C). Thus, there might be a minor role for TRPV3 in thermal selection behavior on the 129S6 background. However, this channel does not appear to be a dominant contributor to this process.

We also assayed acute thermal nociception in TRPV3 knockout 129S6 mice. As with mice on the C57BL6 background, knockout mice responded similarly to wild-type controls in the tail immersion (50°C, 52°C), hot plate (52.5°C, 55°C) and the radiant paw-heating assays (Figure 2D). Again, this argues against a major role for TRPV3 in acute thermal nociception.

### Heat Sensitivity of TRPV3/TRPV4 Double Knockout Mice on the C57BL6 Background

To address the possibility that TRPV3 and TRPV4 might act redundantly during thermal selection or heat nociception, we crossed C57BL6 TRPV4 knockout mice with C57BL6 TRPV3 knockout mice to generate mice that were heterozygous at both TRPV3 and TRPV4 loci. Wild-type and TRPV3/TRPV4 double knockout homozygotes were generated from these mice and their progeny, and assayed for heat sensitivity.

**Figure 3:**
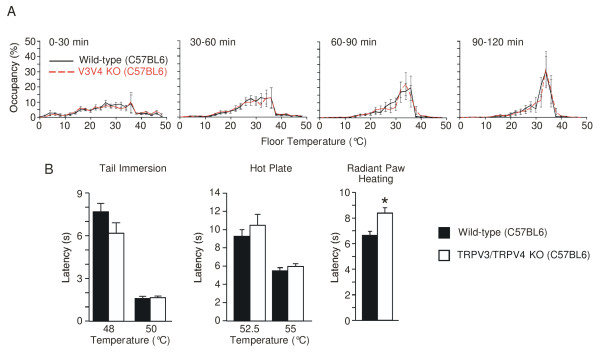
**Temperature preference and heat-evoked nociceptive behavior of TRPV3/TRPV4 double knockout mice on the C57BL6 background**. (A) Thermal preference behavior of freely moving wild-type (black solid line, n = 12) and TRPV3/TRPV4 double knockout (V3V4 KO, red dashed line, n = 10) mice on a floor temperature gradient of 0.8°C to 48.8°C over 120 minutes. Percent of time spent (mean ± SEM) within the indicated 2°C bins was monitored during the indicated time periods. (B) Heat-evoked acute nociceptive behavior of wild-type (filled bars) and TRPV3/TRPV4 double knockout (open bars) mice. Latency to response was measured in the tail immersion (left, n = 12-13) and hot plate (middle, n = 12) assays at the indicated temperatures. Latency to response in the radiant paw heating assay (n = 19-20) was measured during stimulation at a fixed lamp intensity. Data represent mean ± SEM. (*p < 0.05 wild-type vs. knockout, unpaired t-test).

**Figure 4:**
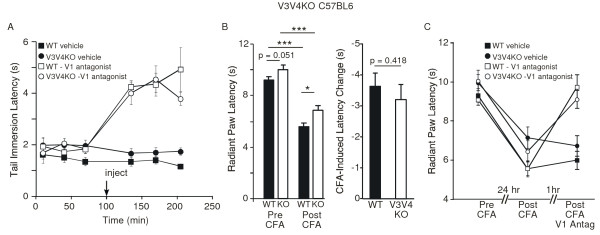
**Effect of TRPV1 antagonism on acute and inflammatory heat-evoked nociceptive behavior of TRPV3/TRPV4 double knockout mice on the C57BL6 background**. (A) Effect of TRPV1 antagonist (JNJ-17203212, 40 mg/kg, i.p., open symbols, n = 7 per genotype) or vehicle (filled symbols, n = 7 per genotype), administered at the indicated time, on tail withdrawal latencies from a 50°C water bath in naïve wild-type (squares) and TRPV3/TRPV4 double knockout (circles) mice. (B) Ipsilateral paw withdrawal latencies to radiant heat in naïve wild-type (filled bars) and TRPV3/TRPV4 double knockout (open bars) mice prior to and 48 hr after intraplantar injection of complete Freund's adjuvant (CFA, 50% in saline) in the left hindpaw (10 μl) (wild-type, n = 30; TRPV3/TRPV4 double knockout, n = 32). (C) Effect of TRPV1 antagonist (JNJ-17203212, 40 mg/kg, i.p., open symbols) or vehicle (filled symbols), on post-CFA paw withdrawal latencies in the wild-type (squares, n = 15 per treatment) and TRPV3/TRPV4 double knockout (circles, n = 16 per treatment) mice shown in panel (B). Data shown represent mean ± SEM.

In the thermal gradient assay, mice deficient for both TRPV3 and TRPV4 exhibited temperature preference behavior indistinguishable from that of wild-type mice across the 2 hr time course, both arriving at the common preferred temperature of ~34°C (Figure 3A). TRPV3/TRPV4 double knockout C57BL6 mice responded similarly to their wild-type counterparts in the tail immersion (48°C, 50°C) and hot plate (52.5°C, 55°C) assays (Figure 3B). In the radiant paw-heating assay, one cohort of TRPV3/TRPV4 double knockout mice showed slightly prolonged withdrawal latencies, compared to wild-type controls (Figure 3B). A trend in the same direction was seen in an independent set of mice (Figure 4B) but not in a third independent experiment (not shown).

### Acute Heat Nociception in TRPV3/TRPV4 Double Knockout Mice with TRPV1 Antagonism

The prominent role of TRPV1 in heat nociception raised the possibility that its presence may hinder our ability to assess additional contributions to this process from TRPV3 and TRPV4. One way to address this possibility would be to functionally neutralize TRPV1 with a TRPV1-selective antagonist, JNJ-17203212 [22]. Using this same strategy, we had previously uncovered an effect of keratinocyte overexpression of TRPV3 on heat nociception [23]. Within 35 min, systemic administration of JNJ-17203212 (40 mg/kg i.p.) markedly increased tail withdrawal latencies at 50°C in wild-type mice, an effect that was sustained for at least 2 hr (wild-type vehicle vs. wild-type antagonist: *p *< 0.001 at 35, 70, and 105 min, n = 7, two-way ANOVA with Bonferroni correction for repeated measures) (Figure 4A). After TRPV1 antagonist treatment, TRPV3/TRPV4 double knockout mice exhibited an increase in latency that was indistinguishable from that of wild type mice (knockout vehicle vs. knockout antagonist: *p *< 0.001 at 35, 70, and 105 min, *n *= 7; wild-type antagonist vs. knockout antagonist: *p *= 0.3967, *n *= 7; two-way ANOVA with Bonferroni correction for repeated measures). Thus, inhibition of TRPV1 does not unmask a prominent role for TRPV3 or TRPV4 in acute heat nociception.

### Inflammatory Heat Sensitivity of TRPV3/TRPV4 Double Knockout Mice with TRPV1 Antagonism

To address whether the contributions of TRPV3 and TRPV4 to thermal nociception might be greater under inflammatory conditions, we inflamed one hind paw of wild-type and TRPV3/TRPV4 double knockout mice via intraplantar injection of complete Freund's adjuvant (CFA, 50% in saline), and evaluated the change in response latency in the radiant paw heating assay. Naive TRPV3/TRPV4 double knockout mice again exhibited a small apparent increase in baseline response latency, compared with controls, although in this experiment that difference did not quite reach statistical significance (p = 0.051, n = 30-32, unpaired t-test). Two days after CFA injection, both wild-type and double knockout mice exhibited significant reductions in thermal response latency (wild-type pre- vs. post-CFA: *p *< 0.0001, *n *= 30-32; knockout pre- vs. post-CFA: *p *< 0.01, paired student's *t*-test). Under these conditions, the difference between wild-type and double knockout mice reached statistical significance (wild-type post-CFA vs. knockout post-CFA: *p *< 0.05, *n *= 30-32, unpaired student's *t*-test). However, the change in response latency following CFA administration was not statistically different between genotypes (Figure 4B). Because TRPV1 has been demonstrated to make a major contribution to inflammatory heat hyperalgesia, we examined the effects of TRPV1 antagonist administration under these conditions. Half of the CFA-treated mice were treated with JNJ-17203212 (40 mg/kg i.p.) and the other with vehicle. Whereas vehicle had no significant effect on latency, the TRPV1 antagonist increased the post-CFA paw withdrawal latency in both genotypes to near the pre-CFA levels (wild-type vehicle vs. wild-type antagonist: *p *< 0.0001, wild-type pre-antagonist vs. wild-type post-antagonist: p < 0.0001; knockout vehicle vs. knockout antagonist: *p *< 0.01; knockout pre-antagonist vs. knockout post-antagonist: *p *< 0.001; *n *= 15 (wild-type) or 16 (knockout), paired student's *t*-test) (Figure 4C). Moreover, TRPV3/TRPV4 double knockout mice exhibited a post-antagonist latency that was statistically no different from that of wild-type controls. Thus, even under conditions of inflammation, we observed minimal impact of TRPV3 and TRPV4 gene disruption on heat nociception.

## Conclusions

In this study, we found that genetic deletion of TRPV3 alone or simultaneous deletion of TRPV3 and TRPV4, had minimal impact on innocuous or noxious heat sensation under naïve conditions, following inflammation with CFA, or when TRPV1 was masked.

Our findings on TRPV3 knockout mice differ from those of a previous study [19], even though both reports examined mice from the same founder line. Several experimental differences between these two studies may have contributed to the discrepant results. The animals used in the Moqrich et al. study were the progeny of intercrossed C57BL6/129J N1 littermates, and their thermosensory phenotypes might therefore have been influenced by inhomogeneous inheritance of non-TRPV3 determinants. In contrast, the mice used in our study were on more homogeneous C57BL6 and 129S6 genetic backgrounds. Large differences in heat nociceptive behavior have been demonstrated among inbred mice of different genetic backgrounds [21]. At least some of this strain dependence has been linked to the calcitonin gene-related peptide locus [19]. Our data indicate that such strain dependence may also extend to innocuous thermosensation. Wild-type C57BL6 mice selected thermal gradient temperatures rather consistently, forming a single occupancy peak at ~32°C, whereas wild-type 129S6 mice distributed themselves more heterogeneously between one peak at ~22°C and another at ~32°C. On the 129S6 background, the absence of TRPV3 did have a modest effect on thermal selection behavior. On the thermal gradient, TRPV3 knockout 129S6 mice more consistently favored a single narrow range around ~22°C. TRPV3 knockout 129S6 mice exhibited a similarly altered pattern of thermal preference in the two-temperature selection task, although the differences were not statistically significant at any individual time point. In this assay, they showed less preference for 32°C over 22°C and a greater preference for 32°C over 37°C, compared with wild-type controls. The fact that these knockout mice also exhibited an apparently stronger preference for 32°C over 19°C than wild-type controls might at first appear paradoxical. However, closer inspection of Figure 2A reveals that, even in the thermal gradient assay, the "shoulder" of the ~22°C peak of the wild-type 129S6 distribution appears to extend to cooler temperatures than that of the TRPV3 knockouts. While such a trend is certainly within the range of variability of the assay, it is tempting to speculate that it might reflect a generally more restrictive thermal preference of TRPV3 knockout 129S6 mice with a peak ~22°C. It is unclear whether this apparent phenotype arises from one or more specific roles of TRPV3 in governing the breadth of thermal tolerance as a sensor of warm temperatures, as opposed to less specific compensatory adaptations. Given the reported link between TRPV3 and heat-evoked keratinocyte release of ATP [20], however, it is interesting to note that C57BL6 mice lacking the adenosine triphosphate-gated ion channel, P2X3, also exhibit a more restricted distribution than wild-type controls on the thermal gradient, although their occupancy, like that of their wild-type controls, is centered in the 32-34°C range [4]. Still, it is worth emphasizing that we saw no restriction of distribution in TRPV3 knockout C57BL6 mice, compared with wild-type C57BL6 controls. Thus, background strain appears to be a key modifier of mouse thermal selection behavior.

The subtle patterns described above emphasize another possible reason for differences between our results and those of Moqrich et al. [19], namely the use of different assay parameters. The thermal gradient extended from 15°C to 55°C in their study, versus 1°C to 49°C in the present study. For the two-temperature discrimination assay, the Moqrich et al. study employed 3 temperature pairs (room temperature vs. room temperature, room temperature vs. 35°C, room temperature vs. 15°C; room temperature = 22-24°C), whereas in our study, the temperature choices were 33°C vs. 37°C, 34°C vs. 28°C, or 35°C vs. 24°C for C57BL6 mice and 33°C vs. 37°C, 32°C vs. 22°C, or 32°C vs. 19°C for 129S6 mice. Moreover, mice were assayed for 10 min in the Moqrich et al. study but for 60 minutes in the present study. Non-thermal (e.g. spatial) cues in the testing environments may also have contributed to the apparently disparate results. Finally, differences in acute nociception assay criteria might also have contributed to the lack of agreement between studies. For example, in the 55°C hot plate test, wild-type mice exhibited an average withdrawal latency of ~13 seconds in the Moqrich et al. study, versus ~7 to 8 seconds in our study. Despite these differences in assay conditions and mouse populations, both studies found, at most, modest phenotypes in thermal selection and heat nociception behaviors in mice lacking TRPV3.

We previously reported that TRPV4 knockout [16] mice prefer slightly warmer floor temperatures in both thermal gradient and two-temperature selection assays, and exhibit longer tail withdrawal latencies to moderately hot temperatures (45°C and 46°C) but not at higher temperatures. Other investigators reported evidence of reduced inflammatory heat hyperalgesia in these same mice [15]. Based on these data, one might have expected TRPV3/TRPV4 double knockout C57BL6 mice to exhibit deficits in innocuous thermosensation and heat nociception that were at least as pronounced as those reported in TRPV4 knockout mice, if not more so. Surprisingly, thermal selection behavior on the thermal gradient was indistinguishable between wild-type and TRPV3/TRPV4 double knockout mice. Furthermore, thermal nociception was relatively unimpaired in these mice, with the exception of a small increase in response latency in the radiant paw heating assay. Although this difference persisted following paw inflammation with CFA, the reduction in latency produced by inflammation was comparable between genotypes. One possible explanation for these discrepancies with prior findings is that double knockout mice have undergone compensatory changes in non-TRPV3/TRPV4 thermosensory mechanisms, either as a consequence of lifelong absence of these channels or as these mutant alleles were propagated across generations. Variability in such compensation, the small magnitude of the knockout phenotype, and/or subtle differences between assay conditions might explain why we were able to measure changes in the radiant paw heating response latency in some cohorts of mice but not others. In this light, it would be interesting to assess the effects of TRPV3- and/or TRPV4-selective antagonists in wild-type mice, or the acute, inducible disruption of both genes.

Our data also indicate that a TRPV1 does not mask major thermosensory roles for endogenous TRPV3 and TRPV4. We previously used a TRPV1 antagonist to unmask thermal hyperalgesia in transgenic mice overexpressiong TRPV3 in keratinocytes [23]. In contrast, TRPV1 antagonism resulted in increases in heat response latencies to comparable levels between wild-type and TRPV3/TRPV4 double knockout mice in the present study, both under naïve conditions and following inflammation with CFA. Taken together, these findings support the notion that endogenous TRPV3 and TRPV4 are largely expendable for heat nociception. Another candidate contributor to TRPV1-independent thermosensation is TRPV2, which is strongly expressed in a subpopulation of medium-to-large diameter sensory neurons and which can be activated by heat at temperatures > 52°C. Although the thermosensory characterization of TRPV2 knockout mice has not been published, the relatively high threshold for activation of this channel by heat argues for consideration of other mechanisms for heat detection at lower temperatures. Regardless of the signaling mechanism(s) involved, residual heat-evoked responses are most likely mediated by neurons that normally express TRPV1, since chemical ablation of the central terminals of these neurons with intrathecal resiniferatoxin [24] has been reported to ablate the vast majority of heat avoidance behaviors in mice.

It remains possible that endogenous TRPV4 or TRPV3 contribute more substantially to temperature sensation in some capacity, but that our behavioral assays are inadequate to evaluate these contributions. It might therefore be worthwhile to devise operant-based behavioral assays or employ non-behavioral readouts to identify any finer influence TRPV3 and TRPV4 might have on thermosensory physiology. For example, electrophysiological recordings from secondary nociceptive neurons or peripheral nerve fibers may be able to tease out subtle deficits in thermosensory coding. It might be best to perform such studies without the confounding influence of TRPV1. Unfortunately, the very close linkage between TRPV1 and TRPV3 loci [25] precludes the generation of TRPV1/TRPV3 double knockouts using standard crosses between single knockout lines, while the hyperthermic effects of TRPV1 antagonists [23,26,27] would impose experimental constraints on a pharmacologically-based strategy. Moreover, classically-defined warmth receptive peripheral neurons have been best-studied in cats, nonhuman primates, and humans [28,29], but have been exceedingly difficult to identify and characterize neurophysiologically in rodents [30]. Another consideration is that since our behavioral evaluations were restricted to males, we cannot exclude the possible influence of gender on TRPV channel thermosensory contribution.

Although TRPV3 and TRPV4 are heat-sensitive, the major endogenous functions of TRPV3 and TRPV4 may involve processes other than temperature perception. For example, it has been demonstrated that both TRPV3 and TRPV4 are involved in different aspects of forming or maintaining the skin permeability barrier, and in the case of TRPV4, temperature has been shown to modulate this process [31-33]. Multiple studies have also implicated endogenous TRPV4 in mechanical hyperalgesia or hypotonicity-induced pain, particularly under inflammatory or nerve-injured conditions [17,18,34-36].

Our results support the notion that TRPV3 and TRPV4 likely make limited and strain-dependent contributions to innocuous warm temperature perception or noxious heat sensation, even when TRPV1 is masked. These findings imply the existence of other significant mechanisms for heat perception.

## Methods

### Mice

TRPV3 +/- mice [19] backcrossed 8 generations onto a C57BL6 background were obtained from Dr. Ardem Patapoutian (Scripps Research Institute, La Jolla, CA) and interbred with one another. TRPV3 +/-, TRPV3 +/+, and TRPV3 -/- offspring were interbred to obtain experimental mice. TRPV3 knockout 129S6 mice were derived by backcrossing TRPV3 +/- C57BL6 mice first with HATRPV3 C57BL6 transgenic mice [23], then over 6 generations with wild-type 129S6 mice (Taconic Labs). TRPV3 +/-, TRPV3 +/+, and TRPV3 -/- offspring were interbred to obtain TRPV3 knockout and wild-type experimental mice. Only progeny negative for the HA-TRPV3 transgene were included in this study. Mice possessing disrupted alleles of both TRPV3 and TRPV4 on a C57BL6 background were generated by crossing TRPV3 knockout mice (backcrossed 8 generations on C57BL6) with TRPV4 knockout mice (backcrossed 5 generations on C57BL6) [16,18]. From the resulting offspring, either heterozygote x heterozygote or TRPV3-/-;TRPV4-/- x TRPV3-/-;TRPV4-/- matings were used to generate TRPV3-/-;TRPV4-/- (TRPV3/TRPV4 double knockout) mice and wild-type controls for experiments. Age-matched male mice at least 8 weeks of age were used in all experiments. Mice were housed in a temperature and humidity controlled environment with a 12/12 hr light/dark cycle. Behavioral assays were conducted during the light phase. Prior to pain behavior tests, animals were acclimated to individual isolation in plexiglass cylinders and to human handling for ~2 hr per day for 1-2 days. The experiments were conducted with the investigators blinded to genotype and antagonist administration. All experiments were conducted according to protocols approved by The Johns Hopkins School of Medicine Animal Care and Use Committee.

### Temperature Gradient Assay

Animals were individually acclimated for at least 30 min in a plexiglass cylinder prior to experimentation. The thermal gradient assay was conducted as described previously [16,18]. Briefly, an individual mouse was placed in a long plexiglass chamber with aluminum floor that is temperature-controlled to form a floor temperature gradient from 0.8 to 48.8°C. Mouse occupancy across the chamber was monitored via infrared sensors for 2 hr without human presence. Time spent in each temperature region was analyzed in 30 min periods and plotted across the 2 hr assay, with correction for differential representation of different temperature bins across the gradient. Where differences in distribution became apparent, they were analyzed using unpaired student's *t*-test within the temperature range of interest. In some cases, as a separate means of analyzing these data, after normalizing for disparity among bin representation, we calculated a preference index for occupancy at temperatures > 29°C vs occupancy at < 29°C according to the following equation:

### Two-Temperature Preference Assay

Prior to experimentation, animals were individually acclimated for 30-60 min in a plexiglass cylinder. The two-temperature preference assay was conducted as described previously [16,18]. An individual mouse was placed in a rectangular plexiglass chamber with a floor consisting of 4 thermally isolated aluminum blocks. Each diagonally opposed block pair was maintained at one of the two test temperatures. To avoid any potential confound of spatial preference or assay order, wild-type and mutant mice were assayed alternately and the configuration of temperature assignment to the blocks reversed every 3 to 4 mice. Occupancy on the blocks was monitored for 60 min via infrared sensors without human presence. Data were analyzed in 5 min periods using ANOVA with Bonferroni correction for repeated measures or divided into 30 min periods and analyzed by unpaired student's t-test.

### Tail Immersion Assay

Following 30 min individual acclimation to plexiglass cylinders, the distal one third of the tail of a mouse gently restrained in a surgical towel was immersed in a waterbath set to 48°C, 50°C, or 52°C. Time to tail withdrawal from the waterbath was measured. A minimum of 3 responses, obtained at 20 min intervals, were averaged for each mouse. Data were analyzed using unpaired student's t-test.

### Hot Plate Test

Following 30 min acclimation to plexiglass cylinders, an individual mouse was placed in the test chamber with the floor temperature set to 52.5°C or 55°C. The time required for the animal to exhibit withdrawal/escape behavior (shaking or biting of the hindpaws, or jumping) was measured. Animals were tested at 20-30 min intervals to avoid sensitization. A minimum of 3 responses, obtained at 20 min intervals, were averaged for each mouse.. Data were analyzed using unpaired student's t-test.

### Radiant Paw Heating and Inflammatory Heat Hyperalgesia

Animals standing on a glass surface were acclimated to loose, ventilated plexiglass testing boxes for at least 2 days prior to baseline testing. Latency to withdrawal of the left hindpaw from a radiant heat stimulus delivered through the glass at a fixed lamp intensity was assayed at least 3 times at 30 min intervals. For inflammation studies, following baseline testing, the left hindpaw was injected intraplantar with complete Freund's adjuvant (CFA, 50% in saline, 10 μl, Sigma, St. Louis, MO) and the mice returned to their cages. Forty-eight hr after CFA injection, mice were assayed again (3 times at 30 min intervals) for post-CFA radiant heat-evoked withdrawal latencies. Data were analyzed using unpaired student's *t*-test for comparisons between genotypes and paired student's *t*-test for comparison of pre- vs. post-CFA latencies in a given genotype.

### Administration of TRPV1 Antagonist

In some experiments, after tail immersion latencies or post-CFA radiant paw heating latencies were recorded, mice were injected with TRPV1 antagonist (JNJ-17203212, 40 mg/kg, i.p)[22,27] or vehicle (1:4:15 Pharmasolv: Cremaphor: 5% Dextrose) and tail or paw withdrawal latencies reassayed at least three times at 30-35 min intervals. In the CFA experiment, latencies measured at 60. 90, and 120 minutes after TRPV1 antagonist or vehicle administration were averaged to calculate post-treatment latencies. In the tail immersion assay, drug vs. vehicle groups were randomly assigned prior to the experiment. In the CFA experiment, mice were assigned to drug or vehicle groups by a third party to ensure comparable post-CFA/pre-antagonist latencies between vehicle and antagonist treatment groups in a given genotype, while maintaining blinding of the investigator performing the assay.

## Abbreviations

TRPV: Transient Receptor Potential; CFA: Complete Freund's Adjuvant; ANOVA: Analysis of Variance

## Competing interests

The authors declare competing financial interests.

## Authors' contributions

SMH designed experiments, conducted behavioral experiments, analyzed data, and wrote the manuscript. XL and YY, conducted behavioral experiments, analyzed data, and reviewed the manuscript, JW conducted behavioral experiments, managed the mouse colonies, and reviewed the manuscript, MJC designed experiments, conducted experiments, analyzed data, and wrote the manuscript. All authors read and approved the final manuscript.
